# Mechanisms of Trx2/ASK1-Mediated Mitochondrial Injury in Pemphigus Vulgaris

**DOI:** 10.1155/2021/2471518

**Published:** 2021-02-23

**Authors:** Bin Wei, Fenghe Li

**Affiliations:** ^1^Department of Dermatology, The First Affiliated Hospital of Chongqing Medical University, 400016 Chongqing, China; ^2^Department of Vascular Surgery, The First Affiliated Hospital of Chongqing Medical University, 400016 Chongqing, China

## Abstract

**Objective:**

Apoptotic events mediated by mitochondrial injury play an important role on the onset of Pemphigus vulgaris (PV). The thioredoxin-2 (Trx2)/apoptosis signal-regulating kinase 1 (ASK1) signaling pathway is considered a key cascade involved on the regulation of mitochondrial injury. Hence, we have investigated the regulatory mechanism of the Trx2/ASK1 signaling in PV-induced mitochondrial injury.

**Methods:**

Serum and tissue samples were collected from clinical PV patients to detect the oxidative stress factors, cell apoptosis, and expression of members from Trx2/ASK1 signaling. HaCaT cells were cultured with the serum of PV patients and transfected with Trx2 overexpression or silencing vector. Changes in the levels of reactive oxygen species (ROS), mitochondrial membrane potential (△*ψ*m), and apoptosis were further evaluated. A PV mouse model was established and administered with Trx2-overexpressing plasmid. The effect of ectopic Trx2 expression towards acantholysis in PV mice was observed.

**Results:**

A series of cellular and molecular effects, including (i) increased levels of oxidative stress products, (ii) destruction of epithelial cells in the skin tissues, (iii) induction of apoptosis in keratinocytes, (iv) reduction of Trx2 protein levels, and (v) enhanced phosphorylation of ASK1, were detected in PV patients. *In vitro* experiments confirmed that Trx2 can inhibit ASK1 phosphorylation, alleviate ROS release, decrease △*ψ*m, and lower the apoptotic rate. Injection of Trx2-overexpressing vectors *in vivo* could also relieve acantholysis and blister formation in PV mice.

**Conclusion:**

The Trx2/ASK1 signaling pathway regulates the incidence of PV mediated by mitochondrial injury.

## 1. Introduction

Pemphigus vulgaris (PV) is one type of autoimmune blistering disease, related to chronic bullous dermatosis [1]. This condition is typically mediated by immunoglobulin G (IgG) autoantibodies which can dramatically affect the skin and mucosa of affected individuals [2]. In this case, many loose and fragile bullae may emerge at the site of the affected skin and mucosa, and the broken bullae form red erosions that can persist throughout the life of the patients. Studies have demonstrated that autoantibodies against desmoglein 1 (DSG-1) and DSG-3, which bind to DSG and then trigger the shrinkage and dissociation of epidermal keratinocytes (KCs), can be produced in PV patients, therefore leading to the formation of epidermal bullae [3]. Moreover, PV pathogenesis is associated with KC apoptosis induced by mitochondrial injury.

A number of studies have confirmed the presence of various types of mitochondria-related antibodies in the serum of PV patients [4, 5]. Such antibodies can directly enter into KCs and specifically recognize/bind a diverse number of receptors located in the mitochondrial surface [4]. Thereafter, these antibodies may lead to mitochondrial injury and initiate mitochondria-mediated host cell apoptosis, acantholysis, and desmosome attack [6]. Thus, neutralizing these antibodies may eliminate the IgG-induced KC dissociation (acantholysis) and vesicular lesions on the skin of PV patients.

The Trx system has crucial regulatory role on the redox status of living cells. The mitochondria-related Trx system consists of three main components: Trx2, Trx reductase-2 (TrxR2), and peroxiredoxin-3 (Prx3) [7]. Apart from regulating the cellular redox status, Trx2 is also able to form a complex and inhibit the activation of ASK1 (apoptosis signal-regulating kinase 1) [8]. Once the levels of reactive oxygen species (ROS) are increased in the cell, the cysteine residues of Trx2 are oxidized, leading to the dissociation and activation of ASK1, which further triggers the cell apoptosis mediated by mitochondrial injury [9]. Therefore, here, we examined whether Trx2/ASK1 signaling could play a decisive role on the mitochondria-induced cell apoptosis during PV progression. The putative mechanism of action (MOA) of the Trx2/ASK1 pathway towards the KC apoptosis, induced by mitochondrial injury in PV patients, was presently investigated. Here, we provide novel insights for the development of a new target for the prevention and treatment of PV.

## 2. Materials and Methods

### 2.1. Tissue, Serum Collection, and Cell Culture

Serum and tissue samples were collected from 10 PV patients treated at the First Affiliated Hospital of Chongqing Medical University, between January and June 2018; 10 healthy people serum were also collected from the same hospital. The titer of antibody in patient's serum and skin tissue against DSG-1 and DSG-3 was shown in Fig. S1A and Fig. S1B. As control, serum samples were also obtained from 10 normal subjects. This study was approved by the Ethics Committee of the Chongqing Medical University. All the patients signed an informed consent to participate in this study. HaCaT cells (human immortalized KCs) were purchased from China Center for Type Culture Collection. HaCaT cells were cultured in 5% CO_2_ at 37°C in regular Dulbecco's Modified Eagle's Medium (DMEM) (Gibco, Invitrogen, Carlsbad, CA, USA) containing low concentration of Ca^2+^ (0.07 mM) and supplemented with 10% heat-inactivated fetal bovine serum.

### 2.2. Terminal Deoxynucleotidyl Transferase-Mediated dUTP Nick-End Labeling (TUNEL) Staining

A TUNEL staining kit (C1086, Beyotime, China) was utilized to detect the KC apoptosis in PV tissues. For this, skin tissues were embedded in paraffin blocks and then sliced to 4 *μ*m thick sections, followed by deparaffinization with xylene. Sections were further incubated with DNase-free proteinase K (20 *μ*g/mL), added dropwise at 37°C for 30 min, and then washed with phosphate-buffered saline (PBS) twice. Later, each section was treated with 50 *μ*L of TUNEL assay solution and incubated in the dark at 37°C for 60 mins. After washing with PBS for 3 times, the sections were mounted using antifluorescence quenching medium, and then observed and photographed under a fluorescence microscope.

### 2.3. Enzyme-Linked Immunosorbent Assay (ELISA)

The content of superoxide dismutase (SOD), catalase (CAT), and glutathione peroxidase (GSH-Px) was determined by ELISA according to the manufacturers' instructions (USCN, Wuhan, China). Specifically, 100 *μ*L of each test sample or standard control was loaded into precoated ELISA plates and incubated at 37°C for 60 min. Subsequently, plate wells were washed with PBST and incubated with 50 *μ*L of enzyme-labeled secondary antibody at 37°C for 30 min. After new round of washes, 50 *μ*L of H_2_O_2_ and 50 *μ*L of TMD were added per well and incubated in the dark at 37°C for 15 min. Thereafter, 50 *μ*L of stop buffer solution was added into each well, and the absorbance at 450 nm was measured using a microplate reader. The enzymatic content of each sample was calculated according to respective standard curves. Moreover, the content of anti-desmoglein-1 and anti-desmoglein-3 in serum and skin tissue of PV patients were also detected by ELISA assay. The ELISA kits of anti-desmoglein-1 (SEA729Hu, Cloud-Clone Corp, Wuhan, China) and anti-desmoglein 3 (SEA444Hu, Cloud-Clone Corp, Wuhan, China) were used for examination. The experiment procedure was following with the instruction.

### 2.4. Western Blotting

Samples of PV skin tissues or cells were lysed in RIPA lysis buffer containing protease inhibitor. Total protein lysates were centrifuged to obtain the supernatant, from which the protein concentration was determined using a BCA kit. The protein content of each sample was subjected to SDS-PAGE, transferred onto membrane, and then blocked. Thereafter, membranes were incubated with respective primary antibodies against Trx2, ASK1, p-ASK1, SOD2, cleaved caspase-3, and GAPDH (diluted at 1 : 800) at 4°C overnight and with horseradish peroxidase-labeled secondary antibodies at room temperature for 2 h. Specific protein bands were developed using an ECL kit and a gel imaging system. Absorbance values were analyzed using Image J.

### 2.5. Immunofluorescence (IF) Assay

PV skin tissues were first sliced using a freezing microtome, heated by microwaving (for antigen retrieval), and then incubated with normal goat serum at room temperature for 15 min. Anti-Trx2 antibody (1 : 500 solution) was added dropwise, and sliced tissues were then incubated at room temperature for 1 hr. Fluorescently labeled secondary antibody (1 : 500 solution) was further added dropwise for an additional one-hour incubation at room temperature. Subsequently, tissue sections were washed with PBS and then incubated with DAPI staining solution, in the dark, at room temperature for 10 min. After one last round of PBS washes, the sections were mounted in antifluorescence quenching medium and then observed/photographed under the fluorescence microscope.

### 2.6. Flow Cytometry

*In vitro* changes in ROS levels and mitochondrial membrane potential (△*ψ*m) were examined by using a flow cytometer. In brief, HaCaT KCs were seeded into a 6-well plate at 5 × 10^4^ cells/well and cultured for 24 hr until adherence. Serum of normal controls and PV patients were then added separately. Cells were further treated with 5 *μ*M JC-1 dye and 5 *μ*M c-H2DCFDA-AM for 60 and 15 min, respectively, followed by flow cytometry analysis. The BD FACSDi Va software was used for data analysis.

### 2.7. Hematoxylin-Eosin (HE) Staining

Formalin-fixed skin samples from PV mice were dehydrated in gradient alcohol, transparentized, and prepared into paraffin-embedded blocks. Afterwards, blocks were sliced into sections, subjected to HE staining, and mounted in neutral balsam. Stained sections were photographed under a light microscope (CX33, Olympus Corporation, Japan).

### 2.8. Statistical Analysis

Data were represented as mean ± standard deviation using the GraphPad Prism 8.0 software (GraphPad software, Inc., San Diego, CA, USA). Paired comparisons were conducted using a Student's *t*-test. Intergroup comparisons were analyzed using one-way ANOVA followed by Tukey's post hoc test. *P* < 0.05 was set as a cut-off for statistically significant differences.

## 3. Results

### 3.1. Mitochondrial Injury Induces KC Apoptosis in PV Patients

**Figure 1:**
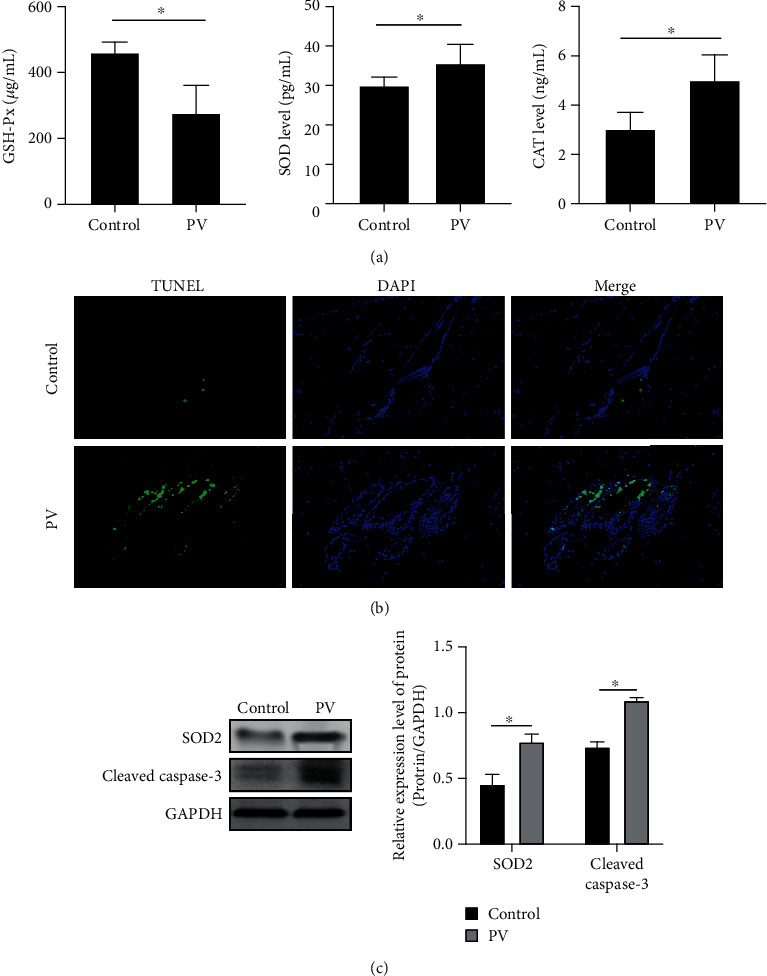
Mitochondrial injury induces KC apoptosis in PV patients. (a) ELISA assay was used to detect the GSH-Px, SOD, and CAT levels; (b) TUNEL assay; (c) Western blot was used to detect the protein expression of SOD2 and cleaved caspase-3. ^∗^*P* < 0.05.

Antimitochondrial antibodies were reported to be generated in PV patients [10]. As a result, these antibodies can destroy the mitochondrial electron transport chain, thus causing the loss of transmembrane potential gradient in the inner mitochondrial membrane and, consequently, increasing the production of oxygen free radicals and weakening the resistance of KCs to oxidative stress [11, 12]. In regard to the oxidative stress products present in the PV patient sera, we currently verified that the content of GSH-Px was lowered, while the levels of SOD and CAT were increased (Figure 1(a)). The increase on oxidative stress products apparently leads to the destruction of epithelial cells in the skin tissue and induces KC apoptosis (Figure 1(b)). Antibodies against SOD2 are common antimitochondrial immunoglobulins typically found in the PV patient sera. In this study, it was observed that both SOD2 and cleaved caspase-3 are highly expressed (Figure 1(c)), suggesting that mitochondrial injury may induce KC apoptosis in PV patients.

### 3.2. Trx2/ASK1 Pathway Is Abnormally Activated in PV Patients due to Mitochondrial Injury

**Figure 2:**
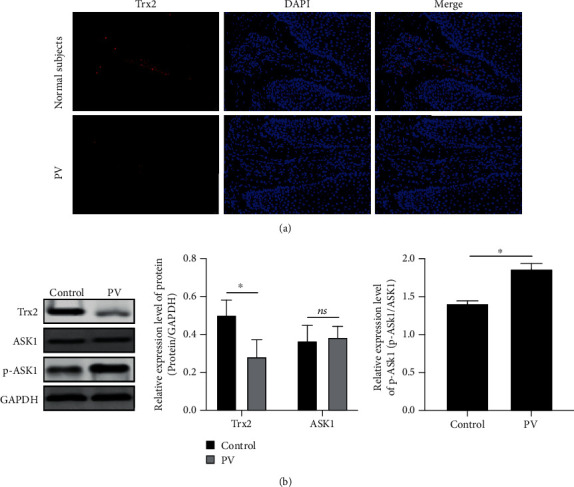
Trx2/ASK1 pathway is abnormally activated in PV patients due to mitochondrial injury. (a) Immunofluorescence assay was used to detect the local expression of Trx2; (b) Western blot was used to detect the protein expression of Trx2 and p-ASK1/ASK1. ^∗^*P* < 0.05.

The mitochondria-specific Trx system is an important player in apoptotic events induced by mitochondrial injury. Elevated ROS levels in PV patients can stimulate the oxidation of cysteine residues in the Trx2 protein, thereby promoting the dissociation of ASK1 from Trx2 complexes and then triggering the cell apoptosis mediated by mitochondrial injury. According to our results, the local expression of Trx2 was reduced in PV patients' lesions (Figure 2(a)). According to the phosphorylation status of downstream proteins, Trx2 protein levels were reduced but, contrarily, the phosphorylation of ASK1 was enhanced (Figure 2(b)). These results indicate that the Trx2/ASK1 signaling cascade might be atypically induced in PV patients due to mitochondrial injury, thus promoting the occurrence and development of PV.

### 3.3. Trx2/ASK1 Signaling Mediates the Serum-Induced KC Apoptosis in PV Patients

**Figure 3:**
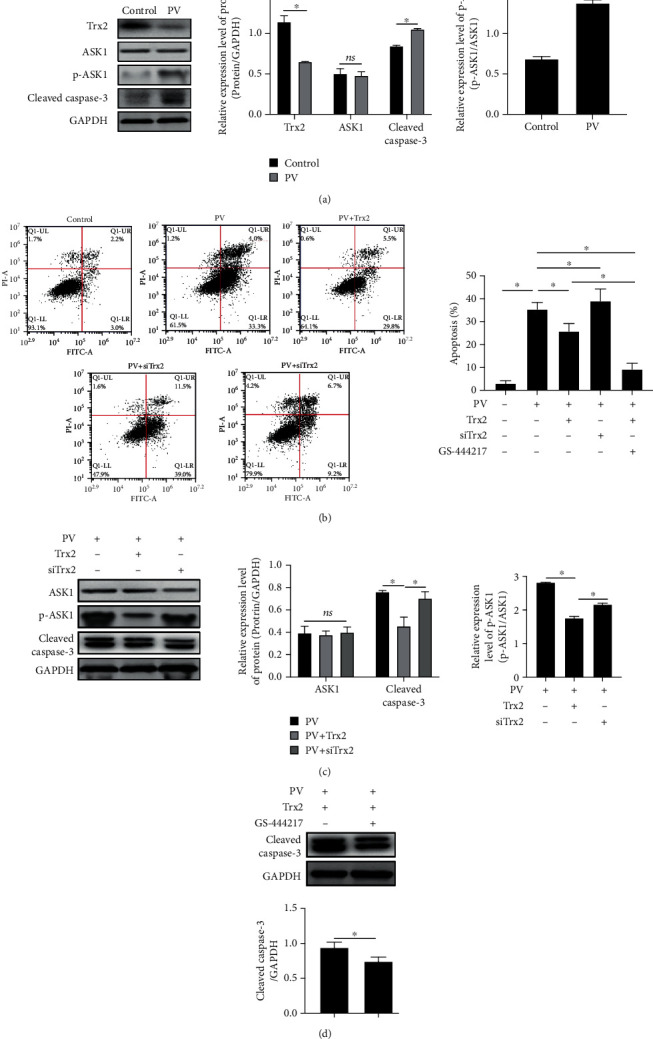
Trx2/ASK1 signaling mediates the serum-induced KC apoptosis in PV patients. (a) Western blot was used to detect the protein expression of Trx2, p-ASK1/ASK1, and cleaved caspase-3; (b) flow cytometry was used to detect the apoptotic rate; (c) Western blot was used to detect the protein expression of p-ASK1/ASK1 and cleaved caspase-3; (d) Western blot was used to detect the protein expression of cleaved caspase-3. ^∗^*P* < 0.05.

HaCaT cells were cultured in media supplemented with sera from PV patients or normal (control) subjects. Western blot analysis indicated that Trx2 levels declined, while the phosphorylation level of ASK1 elevated in the cells culture with sera of PV patients (Figure 3(a)). Moreover, the levels of cleaved caspase-3 as well as the apoptotic rate *in vitro* were both increased (Figures 3(a) and 3(b)). In order to clarify the regulatory mechanism of Trx2/ASK1 signaling, a Trx2-overexpressing vector was constructed and transfected into cells. The apoptotic rate and the levels of cleaved caspase-3 *in vitro* were both reduced in the presence of PV patient sera (Figures 3(a) and 3(b)). In contrast, the apoptotic rate of cells transfected with Trx2 silencing vector was elevated. Additionally, the apoptotic rate in vitro was further reduced upon treatment of Trx2-overexpressing cells with the ASK1-specific inhibitor GS-444217 (Figures 3(c) and 3(d)). These results suggest that the Trx2/ASK1 signaling cascade can modulate cell apoptosis in PV patients.

### 3.4. Trx2/ASK1 Signaling Controls the Serum-Induced Mitochondrial Injury in KCs of PV Patients

**Figure 4:**
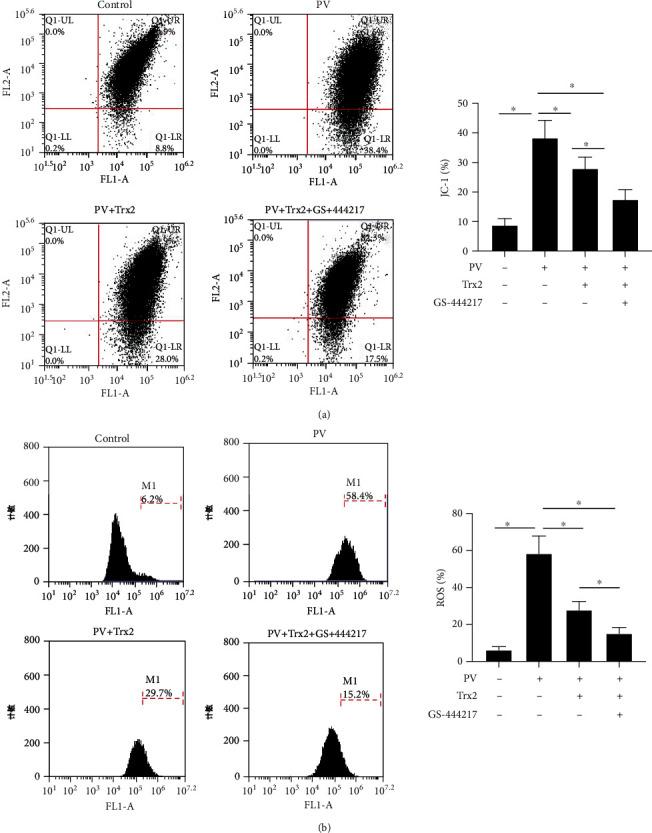
Trx2/ASK1 signaling controls the serum-induced mitochondrial injury in KCs of PV patients. (a) Flow cytometry was used to detect the △*ψ*m marker JC-1; (b) flow cytometry was used to detect ROS. ^∗^*P* < 0.05.

Mitochondrial antibodies are well detected in the sera of PV patients. At the same time, oxidative stress can induce cell apoptosis. In this work, the detection of △*ψ*m and ROS via flow cytometry demonstrated that the △*ψ*m of HaCaT cells, cultured with PV patient sera, was enhanced (Figure 4(a)) and the ROS release was facilitated (Figure 4(b)). Upon Trx2 overexpression, both △*ψ*m and ROS generation were decreased. These indicators decreased even more upon treatment with GS-444217, suggesting that the Trx2/ASK1 pathway might mediate the serum-induced mitochondrial injury in KCs of PV patients.

### 3.5. Trx2 Overexpression Prevents Acantholysis and Reduces KC Apoptosis in PV Mice

**Figure 5:**
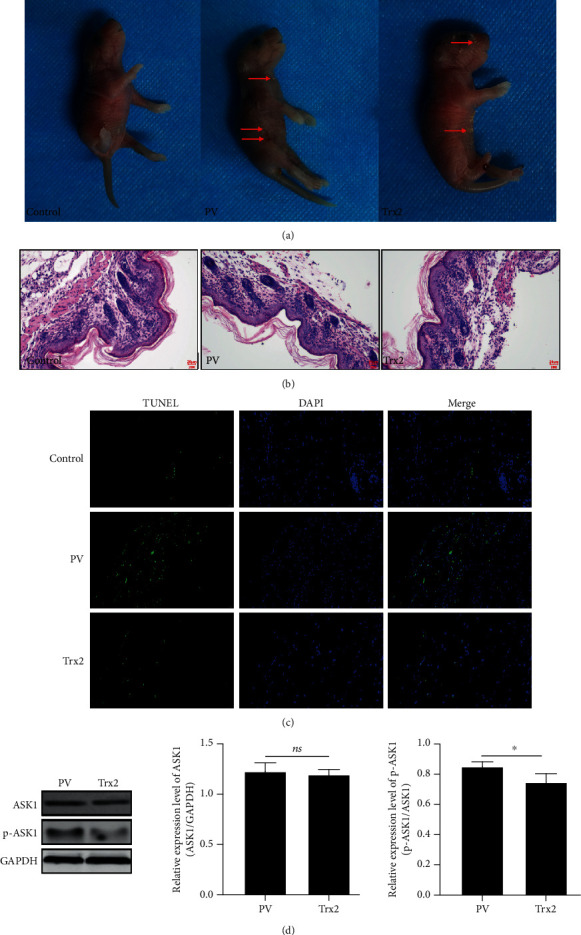
Trx2 overexpression prevents acantholysis and reduces KC apoptosis in PV mice. (a) The photos of PV mice; red arrow showed the lesion site of skin; (b) HE staining of PV mice skin (scale bar = 50 *μ*m); (c) TUNEL assay in PV mice skin; (d) the expression of ASK1 and p-ASK1 in mice skin was detected by Western blot. ^∗^*P* < 0.05.

20 BALB/c neonatal mice were injected with PV patient serum (100 *μ*L/g) subcutaneously (s.c.); then, 10 BALB/c neonatal mice among them were treated with Trx2-overexpressing plasmid (0.1 *μ*g/g) intraperitoneally (i.p.). 10 BALB/c neonatal mice were treated with the same amount of healthy person serum (100 *μ*L/g) as control group. After 48 hours of s.c. injection, acantholysis, intraepidermal blister, and partial exfoliation occurred in the skin lesions and surrounding tissues in PV mice model. However, the epidermal structure was relatively intact, and the acantholysis was alleviated after i.p. injection of Trx2-overexpressing vector (Figure 5(a)). Furthermore, TUNEL assays indicated that Trx2 overexpression could lower the apoptotic rate of KCs in PV mice (Figure 5(b)). At the same time, Trx2 overexpression was also able to repress the phosphorylation level of ASK1 in PV mice (Figure 5(c)).

## 4. Discussion

As an organ-specific autoimmune disease, PV mainly affects the skin and mucosa and jeopardizes patients' lives [13]. Based on the “desmoglein compensation hypothesis,” DSG antibodies currently play vital roles in the onset of PV [14]. Corticosteroid and immunosuppressants are traditional drugs used for PV therapy since they can ameliorate the disease conditions of patients [15]. Still, the side effects of hormonal therapies can increase the mortality rate of the patients. Therefore, seeking for an effective nonhormonal therapy for PV is of seminal significance for the prevention and treatment of this condition. In this study, we confirmed that the oxidative stress products are increased in PV. Due to this effect, epithelial cells from skin tissues are destroyed, and KC apoptosis is induced in PV patients. Besides, the mechanism of injury is apparently correlated with the aberrant activation of the Trx2/ASK1 signaling pathway, which is largely correlated with the mitochondrial injury in PV patients. Moreover, according to a number of in vivo experiments, we validated that Trx2 overexpression can alleviate the serum-induced mitochondrial injury and KC apoptosis in PV patients by inhibiting ASK1 phosphorylation.

Mitochondrial injury is a key pathogenic step in PV [16]. In fact, various mitochondrial antibodies exist in the serum of PV patients. These antibodies can directly enter in KCs to specifically recognize and bind a number of receptors on the surface of the mitochondria. Thereafter, such antibodies induce events linked to mitochondrial injury and initiate the mitochondria-mediated apoptosis, acantholysis, and desmosome attack [4]. More importantly, neutralizing these antibodies can eliminate the IgG-induced KC dissociation (acantholysis) as well as the vesicular lesions on the skin of PV patients.

Trx2 is a key player involved in the regulation of mitochondrial function [17, 18]. Here, we report that the local expression of Trx2 declines within the lesions of PV patients, while the phosphorylation level of its downstream protein ASK1 elevates. These observations indicate that the Trx2/ASK1 cascade is abnormally activated in PV patients affected by mitochondrial injury. Therefore, Trx2/ASK1 signaling appears to be directly related to the incidence of PV.

The mitochondria-specific Trx system includes three main components (i.e., Trx, TrxR, and Prx), and it has crucial regulatory effects on the redox status of cells [19]. In brief, Prx can directly remove ROS, while TrxR can reverse the oxidation status of Trx and thus restore the dual effects of oxidation and reduction. Apart from regulating the cellular redox status, Trx2 is capable of forming complexes with ASK1 and, consequently, to inhibit its activation. ASK1 is a type of serine/threonine kinase that can be activated during oxidative stress [20]. Once ROS levels are increased in the cell, the cysteine residues of Trx2 are oxidized, so that ASK1 is dissociated from Trx2 and activated, thereby triggering apoptotic events mediated by mitochondrial injury [8]. Several studies have indicated that the Trx2/ASK1 signaling pathway plays a decisive role in mitochondria-induced cell apoptosis [21]. Using KCs cultured with PV patient sera, here, we revealed that the levels of Trx2 were diminished, while ASK1 phosphorylation was enhanced. Upon Trx2 overexpression *in vitro*, both △*ψ*m and ROS release were decreased, the apoptotic rate was lowered, and the expression of downstream proteins was inhibited. In order to elaborate the protective effect of Trx2 against PV, a PV mouse model was first established. After administration of Trx2 plasmid in PV mice, we notice that acantholysis was relieved and apoptotic rates were decreased. Taken together, Trx2 appears to inhibit ASK1 phosphorylation to further improve the mitochondrial injury and then reduce the apoptosis rate in PV, thus preventing the progression of this pathological condition.

## Data Availability

The datasets used and/or analyzed during the current study are available from the corresponding author on reasonable request.

## Supplementary Materials

Supplementary Materials Fig. S1: the clinic information of PV patients. (A) The content of DSG-1 and DSG-3 in serum detected by ELISA. (B) The content of DSG-1 and DSG-3 in skin lesion tissue detected by ELISA; *n* = 10 and ^∗^*P* < 0.05. (c) The immunohistochemistry results of DSG-1 and DSG-3 in skin tissue of PV patients and healthy people.
